# Acrolein—an α,β-Unsaturated Aldehyde: A Review of Oral Cavity Exposure and Oral Pathology Effects

**DOI:** 10.5041/RMMJ.10251

**Published:** 2016-07-28

**Authors:** Dror Aizenbud, Itay Aizenbud, Abraham Z. Reznick, Katia Avezov

**Affiliations:** 1 Department of Orthodontics and Craniofacial Anomalies, School of Graduate Dentistry, Rambam Health Care Campus, Oral Biology Research Laboratory, Technion–Ruth and Bruce Rappaport Faculty of Medicine, Haifa, Israel; 2 Hebrew University, Hadassah, School of Dental Medicine, Jerusalem, Israel; 3 Department of Anatomy and Cell Biology, Ruth and Bruce Rappaport Faculty of Medicine, Technion, Haifa, Israel

**Keywords:** Acrolein, cigarette smoke, oxidative stress, α,β-unsaturated aldehydes

## Abstract

Acrolein is a highly reactive unsaturated aldehyde widely present in the environment, particularly as a product of tobacco smoke. Our previous studies indicated the adverse consequences of even short-term acrolein exposure and proposed a molecular mechanism of its potential harmful effect on oral cavity keratinocytic cells. In this paper we chose to review the broad spectrum of acrolein sources such as pollution, food, and smoking. Consequently, in this paper we consider a high level of oral exposure to acrolein through these sources and discuss the noxious effects it has on the oral cavity including on salivary quality and contents, oral resistance to oxidative stress, and stress mechanism activation in a variety of oral cells.

## INTRODUCTION

### Environmental Distribution of Acrolein and the Oral Cavity

The oral cavity is a unique anatomic structure, characterized by the juxtaposition of soft and hard tissues which are constantly exposed to various endogenic and environmental factors influenced by personal priorities and habits. Its inner mucosal surface is a dynamic habitat that is prone to various physical, inflammatory, immunologic, and malignant injuries.

Acrolein, the simplest α,β-unsaturated aldehyde (systematic name: propenal), is a clear colorless-to-yellowish flammable liquid (at room temperature), with a molecular formula of C_3_H_4_O (CH_2_=CH-CHO). It is extremely acrid with a burnt, sweet, pungent, choking odor that occurs naturally in the environment as a result of combustion of wood. According to Environmental Protection Agency classification, acrolein is a high-priority air and water toxin which irritates mucous membranes.^1^

The general population is exposed to acrolein primarily by inhalation of air, especially indoor air containing environmental tobacco smoke.^2^ Acrolein is a highly reactive compound that rapidly binds to the sulfhydryl groups of proteins and other cellular molecules. Human and animal studies indicate that the primary target of acrolein toxicity following inhalation and oral and dermal exposure is the tissue at the site of contact, as demonstrated by irritation in the respiratory and gastrointestinal tracts, eyes, and skin. α,β-Unsaturated aldehydes are present in healthy subjects’ saliva and airway secretions in low-micromolar concentrations and have been found to elevate up to 10-fold in heavy smokers.^3^,^4^

Owing to its ubiquitous presence in the environment and high reactivity, toxic effects of acrolein on various cells and organs have been extensively studied. The effects of acrolein have been most extensively investigated following exposure by inhalation. Oral cavity tissues are the first to encounter the inhaled air and cigarette smoke, and their responses to harmful stimuli are critical in maintaining local homeostasis. Hence, considering the highest level of acrolein exposure through food substances and smoking and its potential chronic toxicity,^5^,^6^ we aimed to review further the oral cavity exposure and the noxious effect of acrolein in oral pathology, presented in our group’s recently published studies.

## ACROLEIN SOURCES

### Environmental Pollutant

Acrolein is an important pollutant widely distributed in the environment as a major by-product of incomplete combustion of organic matter. It is also produced by photochemical oxidation of hydrocarbons of organic pollutants in the atmosphere.^7^ An estimated minimum of 218 tons of acrolein is released yearly to the atmosphere from anthropogenic sources involving fermentation and ripening processes, industrial waste incinerators, furnaces, fireplaces, power plants, combustion of polyethylene plastics, cigarette smoke, overheated cooking of food and oils, and release of volatile components in forest manufacturing processes.^8^,^9^ As a broad-band biocide, acrolein is intentionally released into the environment as a herbicide and an algaecide in water circuits, irrigation canals, drainage ditches, processing waters, cooling water towers, and water treatment basins to control the growth process of aquatic plants. The main microbiocidal “non-pesticidal” use of acrolein is the active ingredient in a product used by oil companies to cleanse hydrogen sulfide in order to control sulfide-producing bacteria and remove hydrogen sulfide and iron sulfide from oil production and injection wells. Acrolein can also solubilize ferrous sulfide deposits that obstruct wells, tanks, and barrels. Furthermore, acrolein has been known to be used as a component in military poison gas mixtures.^2^

The production of acrolein and its use as biocide result in its release to the environment, with the vast majority emitted to the air. In air, acrolein primarily undergoes degradation by means of reactions with photochemically generated hydroxyl radicals in the troposphere, where its half-life is 15–20 hours.^7^ In water and soil, volatilization and degradation are presumed to be the main removal process. In water, acrolein undergoes reversible hydration to form 3-hydroxypropanal, which undergoes aerobic biodegradation that occurs optimally in acclimated cultures.^10^ The estimated half-life in natural unsterilized water is 30–50 hours.^11^ The degradation of acrolein in soil is believed to occur through hydration, biodegradation, and irreversible binding to organic components in soil. The overall reactivity-based half-life of acrolein in soil is estimated to be between 30 and 100 hours.^12^

Exposure of the general population to acrolein occurs primarily by way of atmospheric contact.^1^ Humans’ predominant environmental exposure is by means of inhalation of cigarette smoke or automotive exhaust. The US Environmental Protection Agency reported mean ambient acrolein concentrations of 14.3 μg/m^3^ (6.2 ppb), ranging from 8.2 to 24.6 μg/m^3^ (3.6 to 10.7 ppb), based upon data from 1961 to 1980.^1^ Concentrations in indoor air may exceed outdoor levels 2- to 20-fold.^2^ Populations residing near waste disposal sites or municipal landfills might be subject to higher than average levels of acrolein in the air since acrolein is volatile, and also via drinking water obtained from groundwater wells. However, exposure to acrolein in drinking water is not considered to be a grave problem for these populations due to the volatilization of acrolein from soils and the retention or degradation of acrolein in soils.^7^

### Acrolein Derived from Cigarette Smoke

Human exposure to a large amount of acrolein is through cigarette smoke. Acrolein is present in very high concentrations in the vapor phase of all cigarettes, and its levels vary up to 10-fold between high-tar and ultralow-tar cigarette smoke extracts. It constitutes 50%–60% of the total vapor phase electrophiles.^13^ The International Agency for Research on Cancer^14^ noted that acrolein concentrations in smoke from various cigarettes ranged from 3 to 220 μg/cigarette. Jones^15^ reported concentrations of acrolein in mainstream smoke (defined as smoke that is directly exhaled from the smoker) ranging from 10 to 140 μg/cigarette and estimated concentrations in sidestream smoke (defined as smoke emitted from the smoldering tobacco between puffs) in the range of 100–1,700 μg/cigarette. Acrolein levels between 2.3 and 275 μg/m^3^ have been reported in smoky indoor environments such as bars and restaurants.^14^

The respiratory tract is commonly exposed to a range of α,β-unsaturated aldehydes from cigarette smoke exposure. It was estimated that, during cigarette smoking, acrolein concentrations on the airway surface may be as high as 80 μM.^16^ α,β-Unsaturated aldehydes are present in saliva and airway secretions in low-micromolar concentrations in healthy subjects and are elevated up to 10-fold in heavy smokers.^17^,^18^ It has been reported that inhalation of acrolein as a result of smoking may be a significant contributor to serious lung injury and death.^7^ Inhaled acrolein can be absorbed and may induce systemic effects by increasing platelet activation as a contributing factor to the prothrombotic risk in humans and may induce atherosclerosis and coronary artery disease.^19^ Moreover, human endothelial cells are particularly sensitive to acrolein. Acrolein adducts may cause systemic endothelial cell dysfunction and atherosclerosis.^20^

### Acrolein Derived from Food

Humans are exposed to acrolein by the ingestion of many foods.^21^,^22^ Such foods include fruits (raspberries, grapes, strawberries, and blackberries), vegetables, fish, caviar, doughnuts, cocoa beans, and some types of cheeses. Acrolein can be formed in the different food-processing stages from amino acids, animal fats, or carbohydrates. In fermentation, ripening, and heating and/or overheating, acrolein is formed during the Maillard reaction as a result of the conversion of amino acids and the oxidative deamination of polyamines.^23^–^25^

Acrolein was detected in non-alcoholic beverages such as coffee and tea.^26^ It also may be produced as an unwanted by-product during alcoholic fermentation or during the storage and maturation of alcoholic products and therefore might be detected in spirits and wines.^27^ Acrolein was detected in the emissions of varieties of heated cooking oils, e.g. during frying and deep frying, and therefore French fries present significant contents of acrolein.^28^ In addition acrolein is produced from thermal degradation of cellophane and polystyrene thermoplastics, which are used for food packaging.^29^

The World Health Organization (WHO) working group determined a tolerable oral acrolein intake of 7.5 μg/kg bodyweight/day.^21^ However, a reliable estimate of the acrolein exposure via food and water is virtually impossible.

### Endogenous Acrolein Production

Acrolein is produced endogenously as well. One important intrinsic source is as a by-product of fragmentation of polyunsaturated fatty acids during lipid peroxidation (LPO). Lipid peroxidation is an oxidative degradation of lipids, such as following free radical damage to cell membranes. Numerous studies have revealed that LPO products are associated with the development of inflammation-related diseases, such as chronic obstructive pulmonary disease (COPD), and vascular diseases including atherosclerosis, Alzheimer’s disease, and stroke.^30^ The end-products of LPO are primarily reactive aldehydes, such as malondialdehyde (MDA) and 4-hydroxynonenal (HNE). In addition, the excretion of HPMA (S-(3-hydroxypropyl) mercapturic acid), a significant LPO degradation product and acrolein metabolite, is elevated in the urine of smokers to a level about twice that of non-smoking subjects.^31^

Another source of intrinsic acrolein is the metabolism of the amino acids methionine and threonine. The conversion of threonine into acrolein was found to be mediated by myeloperoxidase (MPO), an enzyme present in human neutrophils that plays a role in killing bacteria and other pathogens. In humans MPO-mediated oxidation of threonine can yield acrolein under conditions of acute oxidative stress, such as myocardial infarction and stroke.^32^ Polyamines, which are derived from arginine, are also a source of endogenous acrolein.^33^

## CHEMICAL REACTIVITY—ACROLEIN CYTOTOXICITY

Among all α,β-unsaturated aldehydes, acrolein is the strongest electrophile, which accounts for its high reactivity with nucleophiles.^34^ Unsaturated aldehyde reaction with cysteine, lysine, and histidine residues of proteins is by means of the Michael addition reaction. This reaction is referred to as “protein carbonylation” as a result of the addition of the aldehydic carbonyl group to the protein, resulting in a change in its chemical structure. Protein carbonyl content is the most general and well-used biomarker of severe oxidative protein damage.^35^ Acrolein adducts created in the thiol side chain of cysteine are considerably more stable than adducts formed by all other α,β-unsaturated aldehydes.^13^ Because acrolein readily reacts by making covalent adducts with sulfhydryl and amino groups of proteins, it is unlikely to distribute systemically, and thus its adverse effects are characterized in terms of cytotoxicity at the site of entry.

It has been suggested that carbonylated proteins are involved in vital cellular functions including energy metabolism, protein synthesis, tissue damages, cytoskeletal integrity, and neuronal plasticity.^36^ Carbonylation at various nucleophilic sites implicated in acrolein cytotoxicity causes oxidative injury and disturbs the cell redox balance following an increased generation of reactive oxygen species (ROS) and glutathione (GSH) depletion.^37^,^38^ Thus the acrolein adduct mediates oxidative damage to cells and tissues as it impairs the structure and function of biomolecules that are considered to be involved in many pathological conditions.^9^ Moreover, it dysregulates major cellular pathways in the process of apoptosis, transcription, cell cycle control, protein biosynthesis, and cell signaling.^39^

Target organs of toxicity of acrolein are primarily the local tissues affected.^21^ Inhalation of acrolein causes irritation and inflammation of the respiratory tract followed by hyperplasia and metaplasia of the respiratory epithelium. Oral exposure causes gastric ulcers or bleeding.^40^ However, the oral cavity as a local anatomic structure is directly exposed to acrolein deleterious toxicity effects by way of increased proteins and DNA adducts, decreased GSH levels, and the impact on cell signaling pathways.

## THE ROLE OF ACROLEIN IN THE PATHOGENESIS OF ORAL INJURIES

### Oxidative Stress

**Figure 1:**
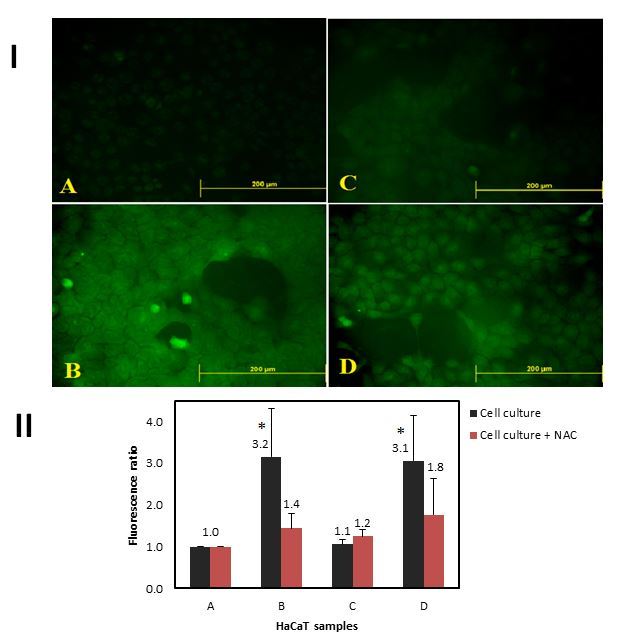
Dichlorofluorescein (DCF) Assay for Cellular Total Oxidation State. I: Fluorescence intensity is proportional to reactive oxygen species and free radicals within the cytosol. A: Air subjected control cell culture; B: cellular oxidative status after a single puff of cigarette smoke; C: after exposure to 200 μmol acetaldehyde; D: after exposure to 1 μmol acrolein. II: Average fluorescence analyses of 3–5 different DCF experiments. Total cellular fluorescence of keratinocytes exposed to cigarette smoke with and without pre-incubation with N-Acetyl Cysteine (NAC). (*=Statistically significant). Reprinted from Figure 4 of Avezov K, et al.,^49^ with permission from Elsevier.

Cellular oxidative stress is implicated in the pathogenesis of many diseases, including strokes, myocardial infarction, and atherosclerosis. In the oral cavity, oxidative stress has been linked with impaired inflammatory response and the onset of periodontal (tooth supporting tissue) destruction.^41^ This is particularly important since there is overwhelming evidence that chronic periodontitis induces a state of systemic inflammation.^42^ Additionally, impaired redox balance is involved in many inflammatory disorders that have oral manifestations, such as lichen planus and pemphigus vulgaris.^43^,^44^ Acrolein changes the redox state of cells by triggering ROS formation or acts as an oxidant in a variety of cells, for instance in retinal cells, bronchial epithelial cells, as well as T cells.^45^–^47^ The reason for the redox change caused by acrolein is probably cellular GSH consumption due to the generation of GSH–acrolein adducts, which prevents its oxidation to glutathione disulfide (GSSG) and subsequent regeneration by the glutathione reductase enzyme.^37^ This has been observed among others in cardiac cells,^18^ pulmonary epithelia,^46^ and oral fibroblasts.^48^ Glutathione depletion could therefore be the mechanism behind cigarette smoke-induced cytotoxicity and could be correlated with the reduced reparative and regenerative activity of gingival and periodontal tissues previously reported in smokers. Systemic elimination of acrolein is performed in the liver by conjugation with GSH,^31^ but in the oral environment, where the exposure to acrolein is direct due to its solubility in saliva, the local elimination by conjugation with glutathione in the cells and the saliva may also be significant. In a unique smoking simulator apparatus direct exposure to cigarette smoke and acrolein caused an increase in cellular oxidative stress in a keratinocytic model of oral exposure.^49^ This was observed concurrently with a decrease in intracellular GSH and ratified that the mechanism of redox change caused by acrolein is probably via cellular GSH consumption in oral epithelial cells as well (Figure 1).

### Adduction to Amino Acids and Cross-linking of Proteins

**Figure 2:**
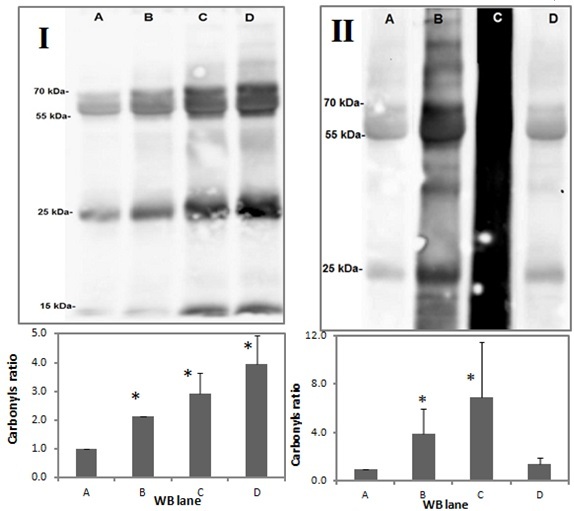
Salivary Protein Carbonylation Assay. I *(upper panel)*: A representative western blot (WB) analysis of total saliva proteins exposed to cigarette smoke. A: untreated control; B: after single puff of cigarette smoke; C: after three puffs of cigarette smoke; D: after nine puffs of cigarette smoke. I *(lower panel)*: Average of densitometric analyses of three to five different WB assays of the same experiment. II *(upper panel)*: A representative WB analysis of the total saliva proteins exposed to aldehydes. A: untreated control; B: acrolein content present in one cigarette (1 μmol); C: acrolein (10 μmol); D: acetaldehyde content present in one cigarette (20 μmol). II *(lower panel)*: Average of densitometric analyses of three to five different WB assays of the same experiment. Reprinted from Figure 3 of Avezov K, et al.,^52^ with permission from Elsevier.

Another biological effect of acrolein is the result of its high reactivity with nucleophilic sites in proteins. In the oral cavity there is a large number of potential protein targets such as enzymes, glycoproteins, and immunoglobulins. In the saliva, inhibition of salivary enzymes such as lactate dehydrogenase (LDH), aspartate aminotransferase (AST), acid phosphatase, and amylase by cigarette smoke was attributed to its unsaturated aldehydic constituents, such as acrolein and crotonaldehyde.^50^,^51^ Dose-dependent carbonyl modifications in salivary proteins were observed when subjected to cigarette smoke and acrolein (Figure 2) but not to acetaldehyde, thus explaining the inhibition in their activities.^52^ Loss of function of salivary enzymes can cause serious damage to their digestive and antimicrobial role. More oral infections are observed in smokers. These include for instance increased likelihood of subgingival infection with certain periodontal pathogens^53^ and oral opportunistic infections such as *Candida albicans* colonization.^54^

The antioxidant properties of the saliva are dependent on the presence of some enzymatic and non-enzymatic antioxidant systems, such as superoxide dismutase enzyme (SOD), catalase, oral peroxidase enzymes, uric acid, and GSH. Salivary GSH content are also depleted due to acrolein exposure^55^ and could further reduce the protective role of saliva against oral oxidative stress.

**Figure 3:**
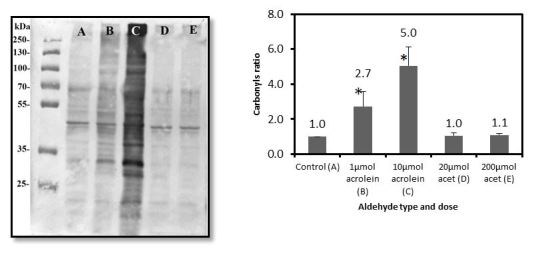
Intracellular Protein Carbonylation Assay. Left: A representative WB analysis of total intracellular proteins exposed to aldehydes. A: Air-exposed control; B: 1 μmol of acrolein (equal to 1 cigarette); C: 10 μmol of acrolein (equal to 10 cigarettes); D: 20 μmol of acetaldehyde (equal to 1 cigarette); E: 200 μmol of acetaldehyde (equal to 10 cigarettes). Right: Intracellular protein carbonyl ratio. Average densitometric analyses of three to five different WB assays of the same experiment. Reprinted from Figure 3, Panels II and III of Avezov K, et al.,^49^ with permission from Elsevier.

Carbonyl protein modifications were observed not only in the salivary fluid, but intracellularly as well, in two types of prevailing oral tissues: fibroblasts^48^ and epithelium (Figure 3). Aldehydes which are readily dissolved in aqueous saliva can easily penetrate cellular membranes and affect cellular contents. A number of stress, cytoskeletal, and redox signal proteins were identified as protein targets in proteomic analysis of bronchial epithelial cells exposed to acrolein.^56^

### Adduction of Acrolein to DNA

The high chemical reactivity of acrolein allows it to react with a variety of macromolecules, including DNA. The reaction of acrolein with a guanosine analog *in vitro* was first described in 1984 by Chung et al.^57^ Since then, the damage by unsaturated aldehydes to DNA was reported in many tissues, especially in lung cells of smokers, where it was suggested to represent a major etiological agent for cigarette smoke-related lung cancer.^58^ A pattern of DNA damage in the p53 tumor suppressor gene produced from acrolein exposure resembles the p53 mutation patterns found in lung cancer.

Cigarette smokers are at substantially greater mortality risk from oral cancer than are non-smokers. Although estimates vary, most studies have reported mortality ratios of about 5–10:1 for never smokers versus smokers. Furthermore, the risk of death from oral cancer is cigarette smoke consumption-related; the more cigarettes consumed daily and the more years one has smoked, the greater the risk. In salivary analyses of oral squamous cell carcinoma patients, the levels of 8-oxoguanine DNA glycosylase, an enzyme involved in base excision repair, were found to be low;^59^ 8-hydroxy-deoxyguanosine (8-OHdG), a widely used indicator of DNA oxidation was found to be higher by 65% along with reduced salivary antioxidants.^60^ Therefore, there is a foundation for the belief that the same mechanisms of oxidative stress induction and DNA damage by acrolein might incite oral cancer as well as lung cancer. Very little is known about the reactivity of acrolein with DNA in oral cells. One of the future objectives of our study group is to investigate and prove this deleterious connection.

### Modulation of Gene Activation by Acrolein

A few transcription factors are regulated by the redox state of the cells. These include oxidative stress-sensing nuclear factor kB (NF-kB) and nuclear erythroid-2 related factor 2 (Nrf2). Nuclear factor kB is involved in the expression of more than 400 genes responsible for the regulation of cellular responses to stimuli such as stress, inflammation, free radicals, radiation, and bacterial or viral antigens. The Nrf2 signaling pathway regulates the cellular redox homeostasis and enzymatic protection against oxidative and electrophilic stress, including the production of enzymes involved in the GSH biosynthesis pathway. Both NF-kB and Nrf2 are present in the cell cytosol in an inactive state, and their release by one of the above-mentioned stimuli permits their translocation to the nucleus, where they regulate gene expression.

Lambert et al.^47^ reported the immunosuppressing effects of cigarette smoke on human T cells. They showed that cigarette smoke extract inhibited transcription of cytokine genes and the production of proinflammatory cytokines, including interleukin-2 (IL-2), IL-10, granulocyte-macrophage colony-stimulating factor, interferon-c, and tumor necrosis factor-a (TNF-a). The cigarette smoke compound responsible for this inhibition appeared to be acrolein, through a direct modification of the DNA-binding domain of the NF-kB pathway.^31^ This NF-kB-mediated gene suppression might be responsible for the reduced periodontal immunologic response to infection found in smokers.^53^

The Nrf2 signaling pathway is also affected by acrolein. It is involved in the cellular protection after exposure to cigarette smoke and aldehydes in the respiratory system and ocular epithelium.^61^ Wu et al.^62^ revealed increased expression of the antioxidant Nrf2-activated enzyme HO-1 in endothelial cells that were exposed to acrolein. Exposure of human lung epithelial (A549) cells to acrolein first depleted 80% of the intracellular GSH and then increased the transcription of c glutamylcysteine synthetase, Nrf2-activated enzyme, resulting in normalization of GSH levels.^63^ The activation of the transcription factors such as Nrf2 pathway in oral cells following cigarette smoke and acrolein exposure has not yet been studied and is currently under extensive investigation by our study group.

## IN CONCLUSION

**Figure 4:**
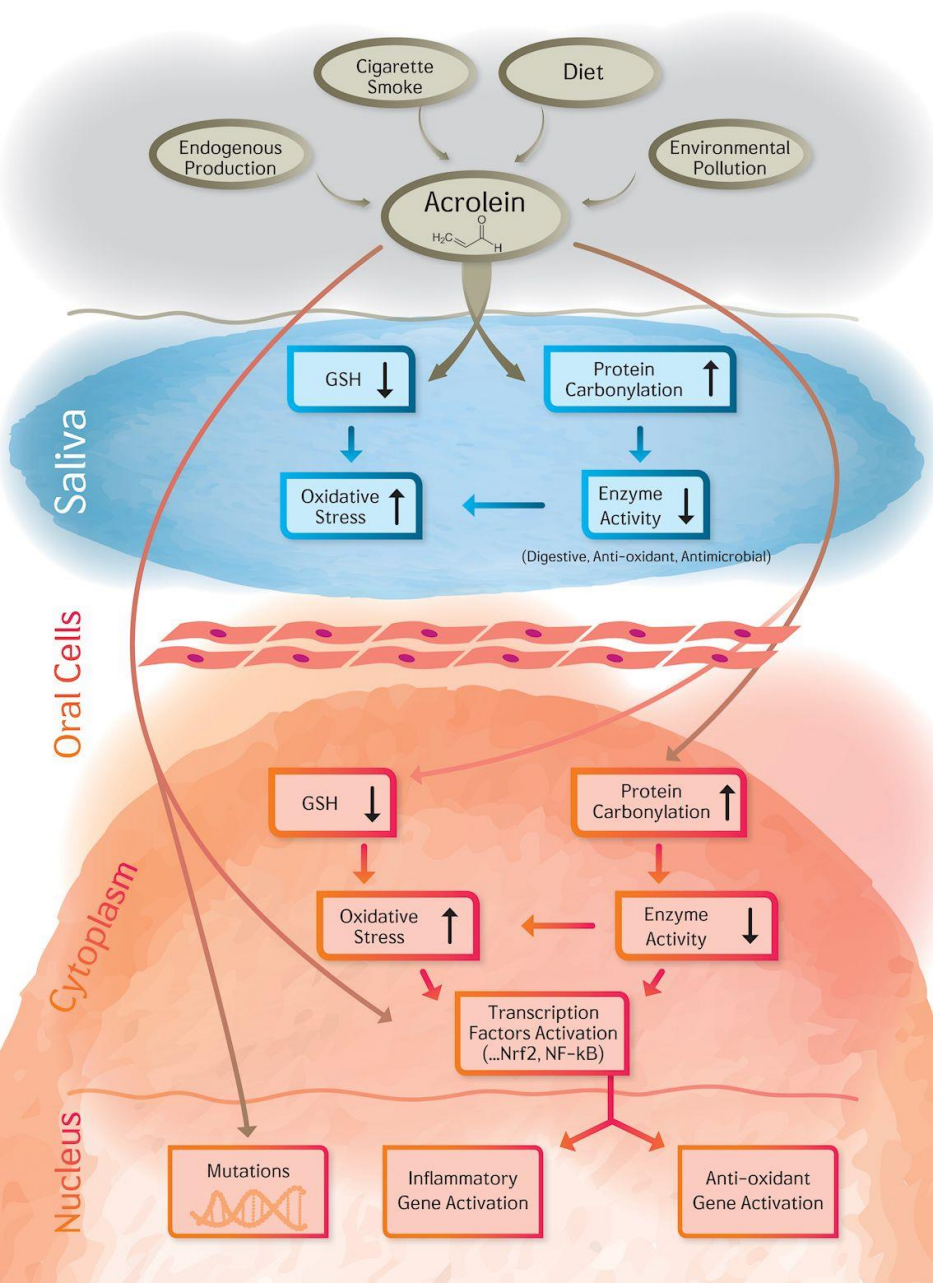
The Summary of Acrolein Effect on the Oral Cavity: The Impact on Saliva and Oral Cells.

The effect of acrolein in the oral cavity is summarized in Figure 4. Due to its high reactivity, acrolein quickly degrades in water, air, and soil. Thus, the direct pathologic damage to the general population from environmental sources is very difficult to establish. However, in oral tissues of smokers, constantly exposed to high levels of acrolein, the toxic effects of acrolein could be the cause of additive damage especially because of its high reactivity at the site of contact. The current paper summarizes for the first time the diverse sources of acrolein and its mechanisms of toxicity in the oral cavity. Further studies should focus on protective pathways, which to date have only been partially revealed.

## Abbreviations

GSH: glutathione
LPO: lipid peroxidation
MPO: myeloperoxidase
NF-kB: nuclear factor kB
ROS: reactive oxygen species

## References

[1] DeWoskin RS, Greenberg M, Pepelko W, Strickland J (2003). Toxicological review of acrolein. Support of Summary Information on the Integrated Risk Information System (IRIS) (CAS No. 107-02-8).

[2] Gomes R, Meek ME, Eggleton E (2002). Concise International Chemical Assessment Document 43. Acrolein.

[3] Andreoli R, Manini P, Corradi M, Mutti A, Niessen WM (2003). Determination of patterns of biologically relevant aldehydes in exhaled breath condensate of healthy subjects by liquid chromatography/atmospheric chemical ionization tandem mass spectrometry. Rapid Commun Mass Spectrom.

[4] Annovazzi L, Cattaneo V, Viglio S (2004). High-performance liquid chromatography and capillary electrophoresis: methodological challenges for the determination of biologically relevant low-aliphatic aldehydes in human saliva. Electrophoresis.

[5] Ismahil MA, Hamid T, Haberzettl P (2011). Chronic oral exposure to the aldehyde pollutant acrolein induces dilated cardiomyopathy. Am J Physiol Heart Circ Physiol.

[6] Weinhold B (2011). Acrolein and neuro disorders. Environ Health Perspect.

[7] Ghilarducci DP, Tjeerdema RS (1995). Fate and effects of acrolein. Rev Environ Contam Toxicol.

[8] Lipari F, Dasch JM, Scruggs WF (1984). Aldehyde emissions from wood-burning fireplaces. Environ Sci Technol.

[9] Aldini G, Orioli M, Carini M (2011). Protein modification by acrolein: relevance to pathological conditions and inhibition by aldehyde sequestering agents. Mol Nutr Food Res.

[10] Bowmer KH, Higgins ML (1976). Some aspects of the persistence and fate of acrolein herbicide in water. Arch Environ Contam Toxicol.

[11] Lijinsky W, Reuber MD (1987). Chronic carcinogenesis of acrolein and related compounds. Toxicol Ind Health.

[12] Mackay JM (1995). Dose selection in in vivo genetic toxicology assays. Environ Mol Mutagen.

[13] Esterbauer H, Schaur RJ, Zollner H (1991). Chemistry and biochemistry of 4-hydroxynonenal, malonaldehyde and related aldehydes. Free Radic Biol Med.

[14] Working Group of the International Agency for Research on Cancer (1979). IARC monographs on the evaluation of the carcinogenic risk of chemicals to humans: some monomers, plastics and synthetic elastomers, and acrolein. IARC Monogr Eval Carcinog Risk Chem Hum.

[15] Jones AP (1999). Indoor air quality and health. Atmos Environ.

[16] Rahman I, MacNee W (1999). Lung glutathione and oxidative stress: implications in cigarette smoke-induced airway disease. Am J Physiol.

[17] Yao H, Rahman I (2011). Current concepts on oxidative/carbonyl stress, inflammation and epigenetics in pathogenesis of chronic obstructive pulmonary disease. Toxicol Appl Pharmacol.

[18] Luo J, Hill BG, Gu Y (2007). Mechanisms of acrolein-induced myocardial dysfunction: implications for environmental and endogenous aldehyde exposure. Am J Physiol Hear Circ Physiol.

[19] Sithu SD, Srivastava S, Siddiqui MA (2010). Exposure to acrolein by inhalation causes platelet activation. Toxicol Appl Pharmacol.

[20] Uchida K (2000). Role of reactive aldehyde in cardiovascular diseases. Free Radic Biol Med.

[21] Abraham K, Andres S, Palavinskas R, Berg K, Appel KE, Lampen A (2011). Toxicology and risk assessment of acrolein in food. Mol Nutr Food Res.

[22] Kuper H, Adami HO, Boffetta P (2002). Tobacco use, cancer causation and public health impact. J Intern Med.

[23] Beauchamp RO, Andjelkovich DA, Kligerman AD, Morgan KT, Heck HD (1985). A critical review of the literature on acrolein toxicity. Crit Rev Toxicol.

[24] Hirayama T (1989). A large-scale cohort study on risk factors for primary liver cancer, with special reference to the role of cigarette smoking. Cancer Chemother Pharmacol.

[25] Lane RH, Smathers JL (1991). Monitoring aldehyde production during frying by reversed-phase liquid chromatography. J Assoc Off Anal Chem.

[26] Feron VJ, Til HP, de Vrijer F, Woutersen RA, Cassee FR, van Bladeren PJ (1991). Aldehydes: occurrence, carcinogenic potential, mechanism of action and risk assessment. Mutat Res.

[27] Moghe A, Ghare S, Lamoreau B (2015). Molecular mechanisms of acrolein toxicity: relevance to human disease. Toxicol Sci.

[28] Srivastava S, Sithu SD, Vladykovskaya E (2011). Oral exposure to acrolein exacerbates atherosclerosis in apoE-null mice. Atherosclerosis.

[29] Zitting A, Heinonen T (1980). Decrease of reduced glutathione in isolated rat hepatocytes caused by acrolein, acrylonitrile, and the thermal degradation products of styrene copolymers. Toxicology.

[30] Lee SE, Park YS (2013). Role of lipid peroxidation-derived alpha, beta-unsaturated aldehydes in vascular dysfunction. Oxid Med Cell Longev.

[31] Stevens JF, Maier CS (2008). Acrolein: sources, metabolism, and biomolecular interactions relevant to human health and disease. Mol Nutr Food Res.

[32] Baker J, Arey J, Atkinson R (2005). Formation and reaction of hydroxycarbonyls from the reaction of OH radicals with 1,3-butadiene and isoprene. Environ Sci Technol.

[33] Sakata K, Kashiwagi K, Sharmin S, Ueda S, Igarashi K (2003). Acrolein produced from polyamines as one of the uraemic toxins. Biochem Soc Trans.

[34] Ishizuka T, Hayashi T, Nakazawa H (2000). Serum vascular endothelial growth factor is a candidate biomarker of renal cell carcinoma in hemodialysis patients. Nephron.

[35] Grimard V, Li C, Ramjeesingh M, Bear CE, Goormaghtigh E, Ruysschaert JM (2004). Phosphorylation-induced conformational changes of cystic fibrosis transmembrane conductance regulator monitored by attenuated total reflection-Fourier transform IR spectroscopy and fluorescence spectroscopy. J Biol Chem.

[36] Mello CF, Sultana R, Piroddi M (2007). Acrolein induces selective protein carbonylation in synaptosomes. Neuroscience.

[37] Kehrer JP, Biswal SS (2000). The molecular effects of acrolein. Toxicol Sci.

[38] Uchida K, Kanematsu M, Sakai K (1998). Protein-bound acrolein: potential markers for oxidative stress. Proc Natl Acad Sci U S A.

[39] Thompson CA, Burcham PC (2008). Genome-wide transcriptional responses to acrolein. Chem Res Toxicol.

[40] Faroon O, Roney N, Taylor J, Ashizawa A, Lumpkin MH, Plewak DJ (2008). Acrolein health effects. Toxicol Ind Health.

[41] Chapple ILC, Matthews JB (2007). The role of reactive oxygen and antioxidant species in periodontal tissue destructtion. Periodontol 2000.

[42] D’Aiuto F, Nibali L, Parkar M, Patel K, Suvan J, Donos N (2010). Oxidative stress, systemic inflammation, and severe periodontitis. J Dent Res.

[43] Sezer E, Ozugurlu F, Ozyurt H, Sahin S, Etikan I (2007). Lipid peroxidation and antioxidant status in lichen planus. Clin Exp Dermatol.

[44] Yesilova Y, Ucmak D, Selek S (2013). Oxidative stress index may play a key role in patients with pemphigus vulgaris. J Eur Acad Dermatol Venereol.

[45] Li X, Liu Z, Luo C (2008). Lipoamide protects retinal pigment epithelial cells from oxidative stress and mitochondrial dysfunction. Free Radic Biol Med.

[46] Grafstrom RC, Dypbukt JM, Willey JC (1988). Pathobiological effects of acrolein in cultured human bronchial epithelial cells. Cancer Res.

[47] Lambert C, McCue J, Portas M (2005). Acrolein in cigarette smoke inhibits T-cell responses. J Allergy Clin Immunol.

[48] Colombo G, Dalle-Donne I, Orioli M (2012). Oxidative damage in human gingival fibroblasts exposed to cigarette smoke. Free Radic Biol Med.

[49] Avezov K, Reznick AZ, Aizenbud D (2014). Oxidative damage in keratinocytes exposed to cigarette smoke and aldehydes. Toxicol In Vitr.

[50] Zappacosta B, Persichilli S, Mordente A (2002). Inhibition of salivary enzymes by cigarette smoke and the protective role of glutathione. Hum Exp Toxicol.

[51] Nagler R, Lischinsky S, Diamond E, Drigues N, Klein I, Reznick AZ (2000). Effect of cigarette smoke on salivary proteins and enzyme activities. Arch Biochem Biophys.

[52] Avezov K, Reznick AZ, Aizenbud D (2014). LDH enzyme activity in human saliva: the effect of exposure to cigarette smoke and its different components. Arch Oral Biol.

[53] Zambon JJ, Grossi SG, Machtei EE, Ho AW, Dunford R, Genco RJ (1996). Cigarette smoking increases the risk for subgingival infection with periodontal pathogens. J Periodontol.

[54] Soysa NS, Ellepola ANB (2005). The impact of cigarette/tobacco smoking on oral candidosis: an overview. Oral Dis.

[55] Zappacosta B, Persichilli S, De Sole P, Mordente A, Giardina B (1999). Effect of smoking one cigarette on antioxidant metabolites in the saliva of healthy smokers. Arch Oral Biol.

[56] Spiess PC, Deng B, Hondal RJ, Matthews DE, van der Vliet A (2011). Proteomic profiling of acrolein adducts in human lung epithelial cells. J Proteomics.

[57] Chung FL, Young R, Hecht SS (1984). Formation of cyclic 1,N2-propanodeoxyguanosine adducts in DNA upon reaction with acrolein or crotonaldehyde. Cancer Res.

[58] Feng Z, Hu W, Hu Y, Tang M (2006). Acrolein is a major cigarette-related lung cancer agent: preferential binding at p53 mutational hotspots and inhibition of DNA repair. Proc Natl Acad Sci U S A.

[59] Shpitzer T, Hamzany Y, Bahar G (2009). Salivary analysis of oral cancer biomarkers. Br J Cancer.

[60] Nagler RM, Klein I, Zarzhevsky N, Drigues N, Reznick AZ (2002). Characterization of the differentiated antioxidant profile of human saliva. Free Radic Biol Med.

[61] Kosmider B, Messier EM, Chu HW, Mason RJ (2011). Human alveolar epithelial cell injury induced by cigarette smoke. PLoS One.

[62] Wu CC, Hsieh CW, Lai PH, Lin JB, Liu YC, Wung BS (2006). Upregulation of endothelial heme oxygenase-1 expression through the activation of the JNK pathway by sublethal concentrations of acrolein. Toxicol Appl Pharmacol.

[63] Tirumalai R, Rajesh Kumar T, Mai KH, Biswal S (2002). Acrolein causes transcriptional induction of phase II genes by activation of Nrf2 in human lung type II epithelial (A549) cells. Toxicol Lett.

